# Associations of urinary phthalate metabolite and replacement chemical concentrations with gestational blood pressure

**DOI:** 10.1210/jendso/bvag158

**Published:** 2026-07-13

**Authors:** Kimberly L Parra, Emma V Preston, Marlee R Quinn, Paige L Williams, Michele R Hacker, Thomas F McElrath, David E Cantonwine, Antonia M Calafat, Julianne Cook Botelho, Blair J Wylie, Camille E Powe, Ellen W Seely, Tamarra James-Todd

**Affiliations:** Department of Environmental Health, Harvard T.H. Chan School of Public Health, Boston, MA 02115, USA; Department of Environmental Health, Harvard T.H. Chan School of Public Health, Boston, MA 02115, USA; Department of Environmental Health, Harvard T.H. Chan School of Public Health, Boston, MA 02115, USA; Department of Epidemiology, Harvard T.H. Chan School of Public Health, Boston, MA 02115, USA; Department of Biostatistics, Harvard T.H. Chan School of Public Health, Boston, MA 02115, USA; Department of Epidemiology, Harvard T.H. Chan School of Public Health, Boston, MA 02115, USA; Department of Obstetrics and Gynecology, Beth Israel Deaconess Medical Center, Harvard Medical School, Boston, MA 02215, USA; Department of Epidemiology, Harvard T.H. Chan School of Public Health, Boston, MA 02115, USA; Division of Maternal Fetal Medicine, Brigham and Women's Hospital, Harvard Medical School, Boston, MA 02115, USA; Division of Maternal Fetal Medicine, Brigham and Women's Hospital, Harvard Medical School, Boston, MA 02115, USA; Division of Laboratory Sciences, National Center for Environmental Health, Centers for Disease Control and Prevention, Atlanta, GA 30333, USA; Division of Laboratory Sciences, National Center for Environmental Health, Centers for Disease Control and Prevention, Atlanta, GA 30333, USA; Department of Obstetrics and Gynecology, Beth Israel Deaconess Medical Center, Harvard Medical School, Boston, MA 02215, USA; Diabetes Unit, Massachusetts General Hospital, Harvard Medical School, Boston, MA 02114, USA; Department of Medicine, Massachusetts General Hospital, Harvard Medical School, Boston, MA 02114, USA; Department of Obstetrics and Gynecology, Massachusetts General Hospital, Harvard Medical School, Boston, MA 02114, USA; Division of Endocrinology, Diabetes, and Hypertension, Brigham and Women's Hospital, Harvard Medical School, Boston, MA 02115, USA; Department of Environmental Health, Harvard T.H. Chan School of Public Health, Boston, MA 02115, USA; Department of Epidemiology, Harvard T.H. Chan School of Public Health, Boston, MA 02115, USA

**Keywords:** blood pressure, hypertensive disorders of pregnancy, gestational hypertension, phthalates, endocrine disruptors, pregnancy

## Abstract

**Introduction:**

Some phthalates and their replacements are endocrine disrupting chemicals (EDCs) found in consumer products and linked to hypertensive disorders of pregnancy (HDP). Few studies have evaluated associations between EDCs and gestational blood pressure.

**Objective:**

To evaluate associations between EDC biomarkers with gestational blood pressure.

**Methods:**

The Environmental Reproductive and Glucose Outcomes study (*N* = 338) measured urinary concentrations of 18 phthalate and replacement metabolites and systolic (SBP) and diastolic (DBP) blood pressure at 3 pregnancy study visits (median: 12, 19, and 26 weeks’ gestation). Individual metabolite concentrations and summary measures based on common source or mechanism (eg, ΣPersonal care products [ΣPCP]) were log_2_-transformed, specific-gravity corrected, and modeled continuously and in quartiles. We estimated covariate-adjusted associations of individual metabolites and, summary measures with blood pressure using linear regression, and with HDP using log-binomial regression, assessing metabolite mixtures using Bayesian Kernel Machine Regression.

**Results:**

HDP prevalence was 12.7%. Higher concentrations of monoethyl phthalate (MEP) and ΣPCP were most consistently associated with SBP and DBP elevations at visit 3. Higher concentrations of MEP (Q4 vs Q1) were associated with 4.59 mmHg higher SBP (95% CI: 0.04, 9.15), and higher ΣPCP (Q3 vs Q1) were associated with 5.78 mmHg (95% CI: 1.59, 9.97). Higher MEP and ΣPCP (Q4 vs Q1) were positively linked to DBP elevations of 4.51 to 4.70 mmHg. Other phthalate biomarkers were associated with blood pressure at specific time points.

**Discussion:**

Certain urinary phthalate biomarkers, particularly MEP and ΣPCP, were associated with higher blood pressure levels in pregnancy, with implications for HDP.

Roughly 10% of all pregnancies in the United States are complicated by a hypertensive disorder of pregnancy (HDP), a condition that is associated with a 2-3-fold increased risk of cardiovascular disease in later-life [1]. HDPs are characterized by elevated blood pressure (≥140/90 mmHg), and the development of additional symptoms related to a spectrum of diagnoses that include gestational hypertension and preeclampsia [2, 3]. Even subclinical elevations of blood pressure may increase the risk of cardiovascular disease after pregnancy [2]. Well-known risk factors for high blood pressure during pregnancy include older maternal age, obesity, genetics, and pre-existing metabolic conditions such as diabetes, among others [1, 4]. In addition, a growing focus has centered on environmental exposures, including endocrine disrupting chemicals (EDCs).

Phthalates are a group of EDCs strongly associated with hormonal dysfunction during the perinatal period [5, 6]. This class of EDCs are found in many consumer products, from personal care products (PCP) to food packaging and plastics [7-10]. Phthalates can be ingested, inhaled, and dermally absorbed [11, 12]. As such, phthalate exposure is ubiquitous with over 95% of the United States population, including vulnerable pregnant women and their developing fetuses, having detectable concentrations of urinary phthalate metabolites [12]. While the mechanisms of how phthalate exposure can affect blood pressure remain to be elucidated, previous studies suggest that phthalate exposure may contribute to alterations in blood pressure and the development of HDP via disruption of blood vessel vasculature formation and remodeling [6].

Recent meta-analyses, including a pooled analysis of observational studies, reported positive associations with specific maternal urinary phthalate metabolite concentrations of elevated blood pressure and HDP risk [13-15]. Specifically, one meta-analysis found positive associations of mono-n-butyl phthalate (MBP) concentrations with elevated systolic (SBP) and diastolic blood pressure (DBP) in both second and third trimesters [14]. Similarly, higher mono-n-benzyl phthalate (MBzP) concentrations in the second trimester were associated with elevated SBP and DBP in the third trimester. In contrast, other metabolites, such as mono-2-ethylhexyl phthalate (MEHP) were found to be negatively associated with SBP and DBP later in pregnancy (second and third trimesters) [14]. Moreover, these meta-analyses and pooled studies reported associations between higher urinary concentrations of MBzP, monoethyl phthalate (MEP), and mono-3-carboxypropyl phthalate (MCPP), with increased odds of HDP [13-15]. Yet, few studies have evaluated phthalate replacement chemicals, including di-2-ethylhexyl terephthalate (DEHTP) and di(isononyl) cyclohexane-1,2-dicarboxylate (DINCH), as mixtures with HDP, SBP, and DBP. Even fewer studies have evaluated more contemporaneous associations, as most studies collected biomarker samples years ago, prior to shifts in exposure patterns driven by changes in the consumer market, including the introduction of phthalate replacement chemicals [11, 16].

In this prospective birth cohort, we aimed to evaluate whether average and time-specific urinary metabolite concentrations of phthalates and their replacement chemicals (ie, DEHTP and DINCH), examined individually and as mixtures were associated with gestational SBP and DBP, as well as HDP. We quantified contemporary concentrations (2016 to 2020) of urinary phthalate metabolites and their replacements, and modeled their summary measures based on correlations, mechanisms, and sources of exposure, given their relevance for increased risk of cardiovascular disease after pregnancy [17].

## Materials and methods

### Study population

The Environmental Reproductive and Glucose Outcomes (ERGO) Study is a prospective, longitudinal birth cohort based in Boston, MA, investigating EDCs and cardiometabolic outcomes during the perinatal period [18]. Briefly, from 2016 to 2020, individuals receiving obstetric care were recruited from 2 local hospitals: Brigham and Women’s Hospital and Beth Israel Deaconess Medical Center. A total of 521 unique pregnancies were recruited into ERGO. After removing 33 pregnancies that were found ineligible after consent (eg, pre-pregnancy diabetes or inability to complete an oral glucose tolerance test due to medical reasons), 488 pregnancies remained eligible into ERGO. To supplement ERGO, 165 unique pregnancies were recruited from the Study of Pregnancy Regulation of INsulin and Glucose (SPRING) based at Massachusetts General Hospital, resulting in a combined total sample size of 653 eligible pregnancies for this analysis (Fig. S1) [19]. The inclusion of SPRING participants bolstered the sample size and was based on complementary study recruitment and data collection for the present analysis. Due to funding disruption, the measurement of endocrine disrupting chemical metabolite concentrations in urine samples from both SPRING and ERGO pregnancy cohorts was delayed. At the time of the present analysis, a total of 323 participants from the ERGO Study and 15 participants from the SPRING study had urinary endocrine disrupting chemical biomarker concentrations. Participants with urinary biomarker concentrations from urine samples collected at 3 time points in pregnancy were harmonized across ERGO and SPRING; details can be found elsewhere [18].

ERGO participants were eligible to participate if, at the time of recruitment, they met the following criteria: gestational age <15 weeks, ≥18 years of age, and planned to receive prenatal care at any of the recruiting hospitals. Individuals were excluded from the study if they had a triplet or higher order of gestation (≥3 fetuses), had a prior diagnosis of type 1 or 2 diabetes, or were unable to complete fasting oral glucose tolerance testing (eg, previous bariatric surgery). Individuals from SPRING were eligible to participate in the ERGO study based on similar criteria, which included being 4-14 weeks gestation at the time of study recruitment. SPRING participants were enrolled between 2016 to 2021 and were comprised of individuals at-risk for gestational diabetes.

### Study visits

Participants were invited to 4 study visits during pregnancy at routine clinical prenatal visits, during which participants completed questionnaires (eg, sociodemographic, lifestyle, behavioral, and stress), and anthropometric assessments. In addition, study participants provided biospecimens including urine, blood, and toenails. Clinical health data were abstracted from medical records by research staff. For the present analysis, we used participant data and urine samples collected from 3 study visits at a median of 11 weeks (visit 1), 19 weeks (visit 2), and 26 weeks of gestation (visit 3). The final analytic cohort included all participants with available data on blood pressure and urinary exposure biomarkers from ≥1 pregnancy visit. We excluded participants with missing biomarker data from all 3 study visits (*n* = 315). Institutional review boards at all involved institutions approved of the protocol. The analysis of de-identified specimens at the Centers for Disease Control and Prevention (CDC) laboratory was determined not to constitute engagement in human subjects’ research.

### Urinary metabolites of phthalates and replacement chemicals

Urine samples were collected in polypropylene urine cups during routine prenatal visits (from 2016 to 2020). During the COVID-19 pandemic participants provided urine samples off-site (eg, homes) as described previously [18]. To minimize any potential risk of contamination, all samples were processed in a biosafety cabinet hood in a fragrance-free and nail polish-free laboratory, prior to aliquoting by trained research staff. Phthalate-free cryovials were used for storage of all urine and blood samples prior to analysis. Research staff measured urinary specific gravity using an Atago 4410 (PAL 10S) Digital Urine Specific Gravity refractometer [20]. Urine samples were then stored at −80 °C before overnight shipment on dry ice for analysis. Urinary metabolite concentrations were used as biomarkers of pregnancy exposure to phthalates and 2 replacement chemicals, DEHTP and DINCH. Laboratory staff at the CDC (Atlanta, GA) quantified urinary concentrations of the following 16 phthalate metabolites: MEP, MBP, MBzP, MCPP, MEHP, mono-hydroxybutyl phthalate (MHBP), mono-isobutyl phthalate (MiBP), mono-hydroxy-isobutyl phthalate (MHiBP), Mono(2-ethyl-5-hydroxyhexyl) phthalate (MEHHP), mono(2-ethyl-5-oxohexyl) phthalate (MEOHP), mono(2-ethyl-5-carboxypentyl) phthalate (MECPP), mono(2-ethyl-5-hydroxyhexyl) terephthalate (MEHHTP), mono(2-ethyl-5-carboxypentyl) terephthalate (MECPTP), monooxononyl phthalate (MONP), monocarboxyoctyl phthalate (MCOP), monocarboxyisononyl phthalate (MCNP), and of 2 DINCH metabolites: cyclohexane-1,2-dicarboxylic acid-mono(carboxyoctyl) ester (MCOCH) and cyclohexane-1,2-dicarboxylic acid-monohydroxy-isononyl ester (MHiNCH). Metabolite concentrations were determined using online solid phase extraction high-performance liquid chromatography-isotope dilution mass spectrometry according to established protocols [21]. Metabolite concentrations below the limit of detection were substituted with values of the $LOD/\sqrt{2}$ [22]. Using specific gravity measured with a handheld refractometer, we calculated specific gravity corrected metabolite concentrations to account for urine dilution using the formula: P_SG_ = P × [(Median Specific Gravity − 1)/(Specific Gravity of Sample − 1)] [23].

As shown in Table S1 [19], in addition to single-metabolite concentrations, we calculated summary measures as the weighted molar sums of metabolites with (1) common parent compounds (ie, sum of di-2-ethylhexyl phthalate metabolites, ΣDEHP) [24], (2) common sources [ie, parent chemicals used in plastics (ΣPlastics) found in food packaging, kitchenware, flooring, medical devices, and toys, and personal care products (ΣPCP) like cosmetics, cleaning or products containing fragrance or parfum] [8, 25], and (3) common mechanisms of hormonal action (ie, ΣAnti-Androgenic and ΣEstrogenic) [26-28].

### Blood pressure measurements and HDP assessment

Our primary outcomes were continuous SBP and DBP (mmHg) at pregnancy visits V1-V3. Participant blood pressure was measured at routine prenatal visits following standard clinical practice and abstracted from electronic medical records by study staff. If clinical blood pressure data were not available on the date of a participant’s study visit, we selected the next available blood pressure data following the study visit. Blood pressure was measured in the seated position with appropriately sized cuffs using an automatic monitor at Massachusetts General Hospital and aneroid sphygmomanometer at Brigham and Women’s Hospital. Beth Israel Deaconess Medical Center also used similar procedures for blood pressure assessment, with the use of either an automatic monitor or aneroid sphygmomanometer. The participants were instructed to sit quietly with the cuff positioned at the level of the heart, both feet resting on the floor while the measurement was made. Our secondary outcome was binary HDP (HDP vs no HDP), where HDP was defined as all incident cases of gestational hypertension (*n* = 24), preeclampsia (*n* = 16), and preeclampsia superimposed on chronic hypertension (*n* = 3), based on clinical diagnosis of HDP during the index pregnancy. Individuals with chronic hypertension were identified via self-reported history of hypertension or clinical diagnosis of hypertension prior to 20 weeks of gestation of the index pregnancy. Participants classified as not having a HDP diagnosis (“no HDP”) were the referent group, including participants with chronic hypertension who did not develop preeclampsia (*n* = 11) (eg, normotensive and chronic hypertensive). Gestational age (weeks) was used to confirm HDP diagnosis emerging after 20 weeks gestation. We did not exclude individuals prescribed anti-hypertensive medications (prior to 20 weeks gestation) (*n* = 14).

### Covariates

Participants self-reported data on sociodemographic, lifestyle, and medical history using questionnaires at each study visit. We considered potential confounders to include in our models based on prior literature and directed acyclic graphs (Fig. S2) [19]. The following variables were considered to be potential confounders: maternal age at enrollment (continuous), college degree (yes/no), race and/ethnicity (non-Hispanic White, non-Hispanic Black, Hispanic, non-Hispanic Asian, Other), visit 1 body mass index (BMI) (kg/m^2^, continuous), and parity at enrollment (nulliparous, parous).

### Statistical analysis

Urinary phthalate metabolite and replacement biomarker concentrations were log_2_-transformed to account for their skewed distributions and to minimize the influence of outliers. We also modeled metabolite concentrations as quartiles (Q1-Q4) to relax the linearity assumption, and to account for metabolites with lower detection frequency. We present the visit-specific distribution of urinary metabolite concentrations as geometric means and SD. Spearman’s rank correlations were used to assess correlations among the metabolites at visit 1. We present summary statistics for demographics and clinical characteristics as means with SD, and counts (*n*) with percentages (%).

Based on previous research indicating that early pregnancy is a sensitive window of exposure for blood pressure and hypertensive disorders of pregnancy, we constructed cross-sectional models to evaluate time-specific associations between urinary phthalate metabolite concentrations and blood pressure outcomes [29-33]. We used multivariable linear regression models to estimate β coefficients and 95% CIs for visit-specific cross-sectional associations of concentrations of each individual metabolite and summary measure with SBP and DBP, separately. To estimate the association of individual metabolite and summary measure levels at visit 1 with HDP risk, we fit separate log-binomial regression models to estimate risk ratios (RRs) and 95% CIs. Model results can be interpreted as the difference in SBP or DBP (linear regression models) or the risk of HDP vs the reference group (log-binomial models) per doubling in metabolite or summary measure concentration. For models where metabolite or summary measure concentrations were modeled as quartiles, model estimates can be interpreted as the difference in mean SBP or DBP comparing each quartile with the lowest quartile as the reference. Final models were adjusted for age, education, race and ethnicity, and baseline BMI.

We also estimated the cross-sectional associations between levels of the overall metabolite mixture with SBP and DBP measurements separately at V1-V3 using non-parametric Bayesian Kernel Machine Regression. Additionally, we used the probit extension of Bayesian kernel machine regression to estimate the associations between the mixture at visit 1 and risk ratio (RR) for HDP [34]. All 18 urinary metabolite and replacement chemical concentrations were log_2_ transformed and scaled for Bayesian kernel machine regression. Final adjusted Bayesian kernel machine regression models were fit with Markov Chain Monte Carlo sampler with 50 000 iterations and checked for convergence using trace plots. We also examined univariate exposure-response functions, to assess the linearity of relationships between metabolite-outcome pairs, while holding all other metabolites at their median concentrations, and calculated posterior inclusion probabilities to determine the relative importance of each metabolite to the overall mixture effect. Analyses were performed using R (version 4.05), including the *bkmr* package for the mixtures analysis [34].

### Sensitivity analyses

Previous studies have shown that BMI could be a confounder or mediator in the pathway between EDCs and HDP [35]. We therefore excluded baseline (V1) BMI to assess the robustness of our results. Additionally, parity may influence both blood pressure and HDP outcomes [36]. To reduce potential bias from selective fertility, as individuals who experience pregnancy complications may be less likely to become pregnant again, we restricted our analysis to nulliparous individuals, as HDP predominately occurs during first pregnancies [37]. To account for multiple comparisons between phthalate biomarkers and their replacement chemicals with blood pressure, we applied the Benjamini–Hochberg false discovery rate correction [38]. We also conducted additional sensitivity analyses excluding (1) those with chronic hypertension (*n* = 11) from our normotensive group, (2) individuals taking anti-hypertensive medication during pregnancy from self-report (*n* = 14), and (3) SPRING participants (*n* = 15) who likely have higher risk of gestational diabetes than ERGO participants. These exclusions resulted in similar associations (data not shown).

## Results

### Participant characteristics

Table 1 shows that in our analytic study population of 338 participants, 43 individuals (12.7%) developed HDP. The mean (SD) age at consent was 32.8 years (4.7), and the average baseline (V1) BMI was 26.5 kg/m^2^ (6.1). Participants included in this analysis were predominantly non-Hispanic White (61%), college-educated (75%), and privately insured (75%). Over half of the participants (*n* = 175, 53%) had given birth before. Compared to those retained in this analysis, the participants excluded from this analytic cohort had a higher baseline BMI (29.2 vs 26.5 kg/m^3^), and were more likely to have HDP (17% vs 13%) and a college degree (82% vs 75%). Table S2 shows participants excluded from this analysis (*n* = 315) [19].

**Table 1:** Characteristics of ERGO study participants in Boston, MA (*N* = 338)

	Mean ± SD or *n* (%)
Age at consent (years)	32.8 ± 4.7
BMI (kg/m^2^) at visit 1	26.5 (6.1)
College degree completed	251 (75.1%)
Race and ethnicity	
Non-Hispanic White	206 (60.9%)
Hispanic/Latinx	49 (14.5%)
Non-Hispanic Black	39 (11.5%)
Non-Hispanic Asian	25 (7.4%)
Other	19 (5.6%)
HDP diagnosis*^a^*	43 (12.7%)
Nulliparous	155 (47.0%)
Insurance	
Private	227 (74.7%)
Medicaid/SSI/Mass Health	72 (23.7%)
Self-pay	5 (1.6%)
Smoked during pregnancy	10 (3.2%)
SBP (mmHg)	
Average visits 1-3	113.7 ± 18.7
Study visit 1	114.2 ± 11.6
Study visit 2	114.1 ± 12.1
Study visit 3	113.1 ± 12.1
DBP (mmHg)	
Average visits 1-3	67.4 ± 6.6
Study visit 1	68.3 ± 8.8
Study visit 2	66.2 ± 8.2
Study visit 3	67.3 ± 8.5

^*a*^HDP includes gestational hypertension, preeclampsia, and preeclampsia superimposed on chronic hypertension.

### Urinary phthalates and DINCH metabolite concentrations

The distribution of visit-specific urinary metabolite concentrations is presented in Table 2. Of the 18 metabolites measured, we observed the highest geometric means (SD) urinary concentrations for MEP, MECPTP, and MEHHTP in pregnancy. MCOCH and MHiNCH had the lowest median concentrations. Most samples had detectable concentrations of the metabolites except for MCOCH (44%), MEHP (56%), and MHBP (66%). As expected, phthalate metabolites derived from the same parent compounds (eg, DEHP metabolites MEHP, MEHHP, MEHOHP, and MECPP) were highly correlated. In contrast, urinary metabolite concentrations originating from different parent compounds tend to show weak to moderate positive correlations with each other at V1; with Spearman rank correlations ranging from −0.06 to 0.62 (Fig. S3) [19].

**Table 2:** Visit-specific specific gravity corrected urinary phthalate and replacement chemical metabolite concentrations (ng/mL) in the ERGO study (2016-2020)

	Geometric mean (SD)				
Metabolite*^a^*	Visit 1 *n* = 310	Visit 2 *n* = 280	Visit 3 *n* = 272	LOD	% above LOD
MEP (Monoethyl phthalate)	26.4 (4.0)	22.3 (4.3)	16.1 (3.1)	1.2	97.9
MBP (Mono-n-butyl phthalate)	5.9 (2.9)	5.1 (3.1)	4.8 (3.0)	0.4	92.0
MHBP (Mono-hydroxybutyl phthalate)	0.9 (2.2)	0.8 (2.2)	0.8 (2.1)	0.4	66.0
MiBP (Mono-isobutyl phthalate)	5.5 (2.4)	4.9 (2.3)	4.5 (2.3)	0.8	94.1
MHiBP (Mono-hydroxy-isobutyl phthalate)	2.3 (2.3)	2.1 (2.2)	2.0 (2.2)	0.4	93.2
MBzP (Monobenzyl phthalate)	2.4 (3.2)	2.0 (3.4)	2.0 (3.7)	0.3	91.8
MCPP (Mono(3-carboxypropyl) phthalate)	1.0 (2.5)	0.8 (2.1)	0.8 (2.2)	0.4	72.5
MEHP (Mono(2-ethylhexyl) phthalate)	1.3 (2.3)	1.2 (2.2)	1.2 (2.3)	0.8	55.9
MEHHP (Mono(2-ethyl-5-hydroxyhexyl) phthalate)	4.0 (2.4)	3.3 (2.3)	3.1 (2.3)	0.4	98.2
MEOHP (Mono(2-ethyl-5-oxohexyl) phthalate)	2.8 (2.5)	2.5 (2.4)	2.5 (2.3)	0.2	98.2
MECPP (Mono(2-ethyl-5-carboxypentyl) phthalate)	5.6 (2.2)	4.6 (2.2)	4.5 (2.0)	0.4	99.4
MEHHTP (Mono(2-ethyl-5-hydroxyhexyl) terephthalate)	14.8 (3.5)	11.5 (3.7)	10.0 (3.1)	0.4	99.7
MECPTP (Mono-2-ethyl-5-carboxypentyl) terephthalate)	62.7 (3.6)	56.6 (4.0)	49.9 (3.2)	0.2	100.0
MONP (Monooxononyl phthalate)	1.7 (2.7)	1.5 (2.9)	1.4 (2.6)	0.4	87.6
MCOP (Monocarboxyoctyl phthalate)	5.1 (2.7)	4.0 (2.7)	3.5 (2.5)	0.3	100.0
MCNP (Monocarboxyisononyl phthalate)	1.3 (2.4)	1.1 (2.3)	1.0 (2.4)	0.2	94.7
MHiNCH (Cyclohexane-1,2-dicarboxylic acid-monohydroxy-isononyl ester)	1.0 (3.1)	1.0 (3.2)	1.0 (3.0)	0.4	71.0
MCOCH (Cyclohexane-1,2-dicarboxylic acid-mono(carboxyoctyl) ester)	0.7 (2.4)	0.7 (2.6)	0.7 (2.6)	0.5	44.4

^*a*^Listed in increasing order of molecular weight.

### Associations between urinary metabolite concentrations—individual and summary measures—with SBP

**Figure 1:**
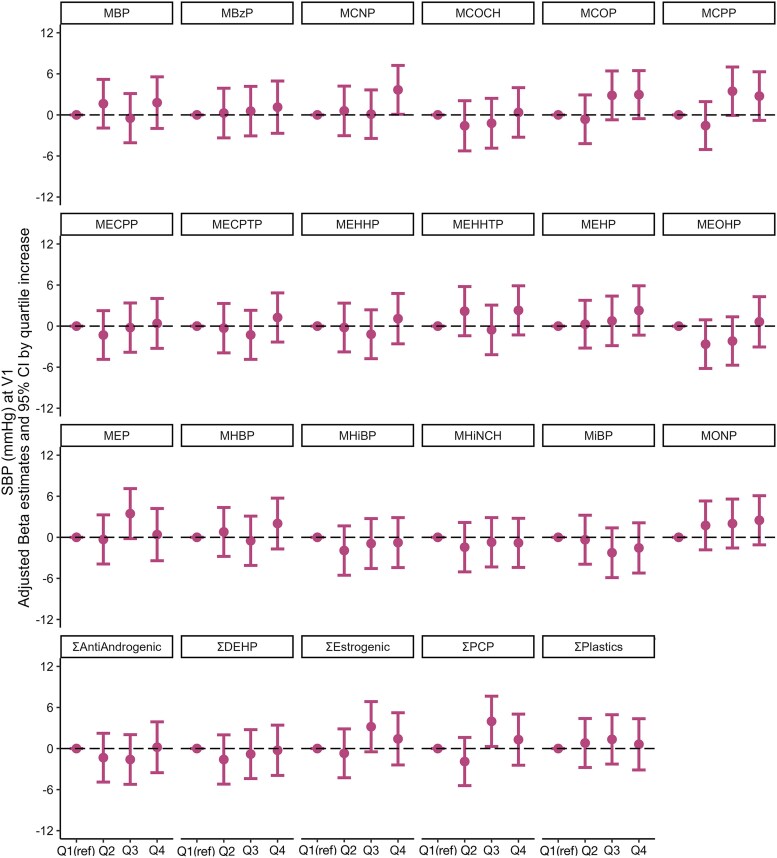
Single pollutant models of SBP at visit 1. Associations of log_2_-transformed and specific gravity corrected urinary biomarker concentrations with systolic blood pressure at visit 1 expressed as difference in mean SBP (mmHg) per quartile increase in biomarker concentration (*n* = 295). Models adjusted for maternal age, education, race and ethnicity, and baseline BMI.

**Figure 2:**
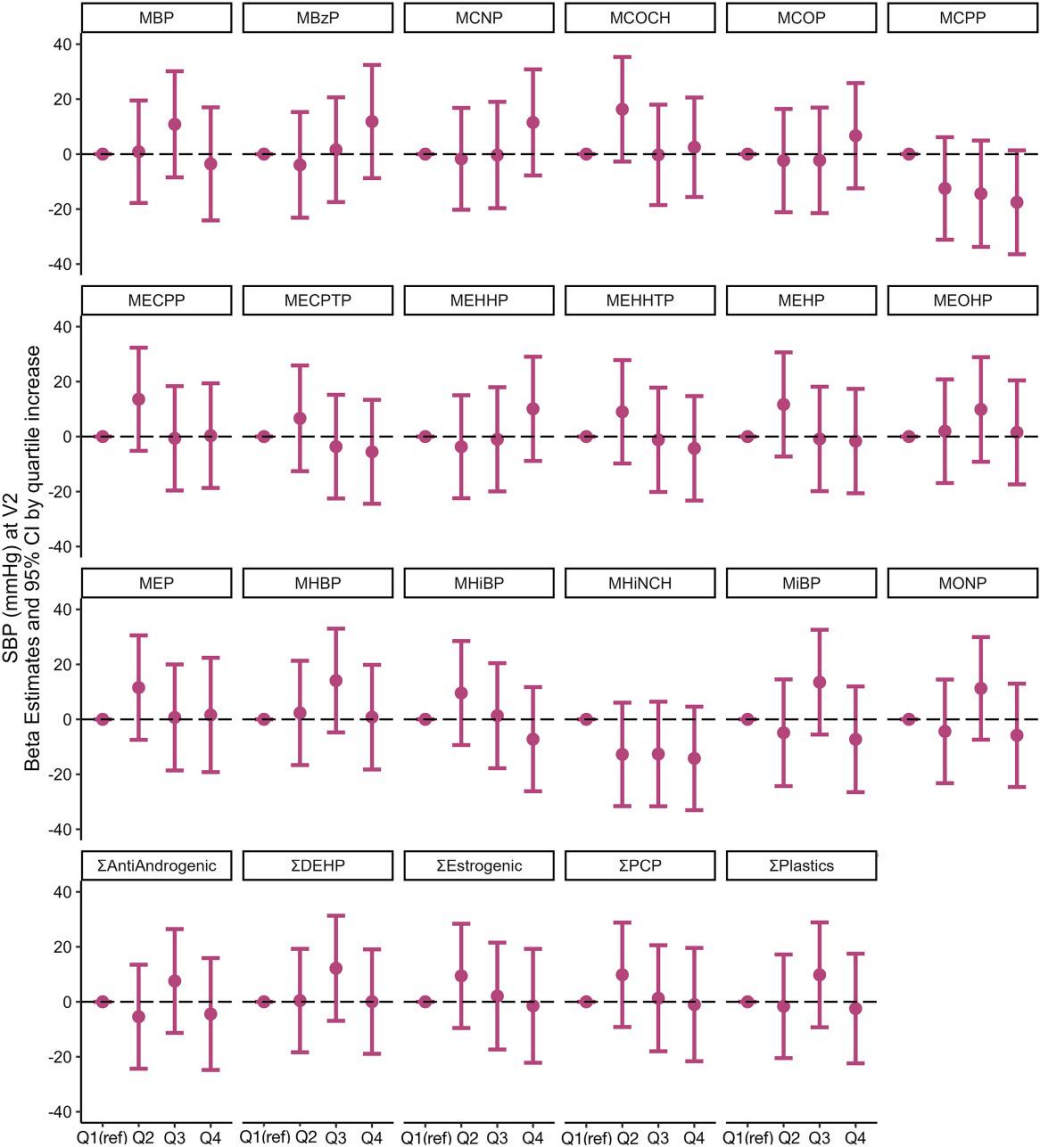
Single pollutant models of SBP at visit 2. Associations of log_2_-transformed and specific gravity corrected urinary biomarker concentrations with systolic blood pressure at visit 2 expressed as the difference in mean SBP (mmHg) per quartile increase in biomarker concentration (*n* = 280). Models adjusted for maternal age, education, race and ethnicity, and baseline BMI.

**Figure 3:**
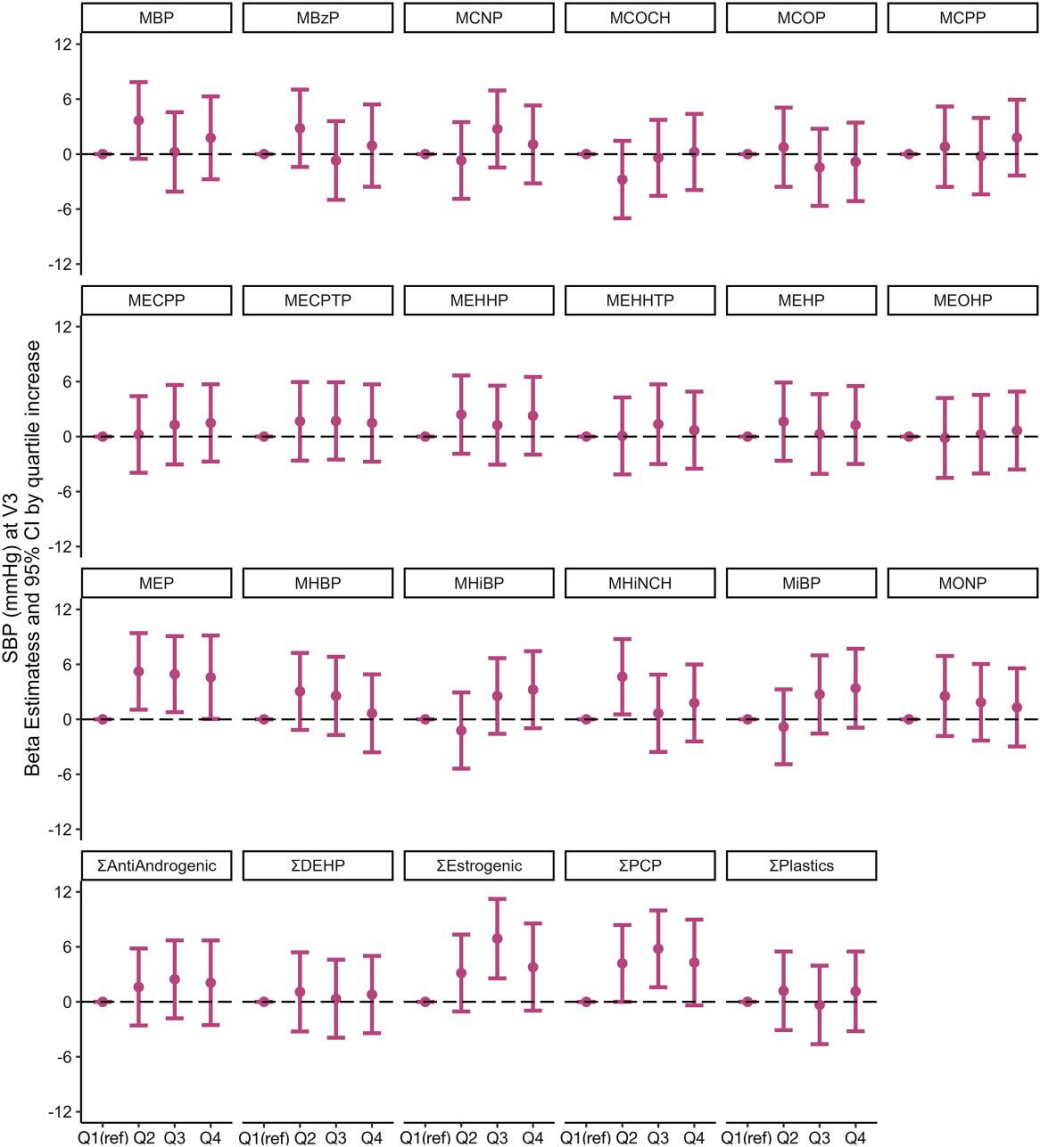
Single pollutant models of SBP at visit 3. Associations of log_2_-transformed and specific gravity corrected urinary biomarker concentrations with systolic blood pressure at visit 3 expressed as the difference in mean SBP (mmHg) per quartile increase in biomarker concentration (*n* = 266). Models adjusted for maternal age, education, race and ethnicity, and baseline BMI.

SBP results from models evaluating individual metabolites and summary measures are shown in Figs. 1-3, Table S3, and Fig. S4 [19]. In adjusted models, we observed that select urinary metabolites and summary measures were cross-sectionally associated with higher SBP levels at V1 and V3, but not V2 (Fig. 2). Specifically, we observed positive associations of certain quartiles of MEP, MCNP, MHiNCH, ΣEstrogenic, and ΣPCP concentrations with SBP at V1 and V3.

At visit 1, participants whose concentrations were in the highest quartile for MCNP, a metabolite of DiNP, had higher mean SBP (Q4 vs Q1 β = 3.66 mmHg, 95%CI: 0.09, 7.23). Similarly, those with ΣPCP in Q3 had higher mean SBP levels (Q3 vs Q1: β = 3.97 mmHg, 95%CI: 0.30, 7.65), but this association was not observed in Q4 (Q4 vs Q:1 β = 1.30 mmHg, 95%CI: −2.44, 5.03) (Fig. 1).

At visit 3, we found stronger and more consistent associations between higher concentrations of MEP and ΣPCP and higher SBP levels, when modeled either as quartiles or as continuous concentrations (Fig. 3). Compared to Q1, the MEP quartiles of Q2, Q3, and Q4 were significantly associated with 4.6 to 5.2-mmHg higher SBP. Each doubling of MEP was also associated with 1.16 mmHg (95%CI: 0.14, 2.17) higher SBP. Similarly, at V3, higher quartile concentrations of ΣPCP (Q2-Q4) were associated with higher mean SBP levels compared to Q1. A doubling of ΣPCP concentration was associated with a 1.43 mmHg (95%CI: 0.16, 2.71) higher mean SBP. Additionally, we observed positive associations between MHiNCH and ΣEstrogenic concentrations with higher mean SBP at V3 (MHiNCH Q2 vs Q1: β = 4.65 mmHg, 95%CI: 0.54, 8.76) and (ΣEstrogenic Q3 vs Q1: β = 6.90 mmHg, 95% CI: 2.56, 11.23).

### Associations between urinary metabolite concentrations—individual and summary measures—with DBP

**Figure 4:**
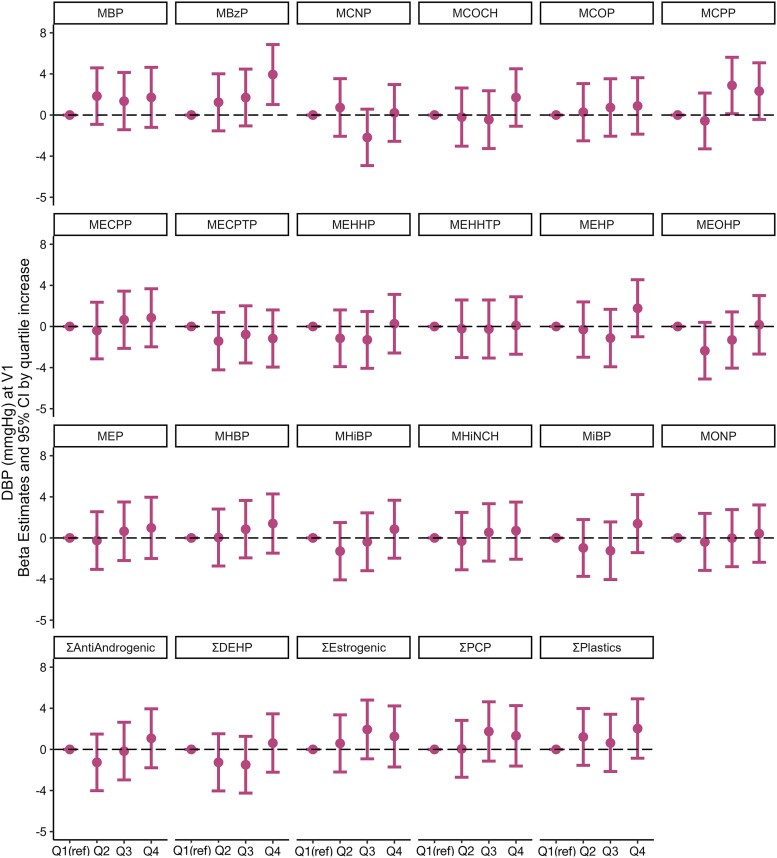
Single pollutant models of DBP at visit 1. Associations of log_2_-transformed and specific gravity corrected urinary biomarker concentrations with diastolic blood pressure at visit 1 expressed as the difference in mean DBP (mmHg) per quartile increase in biomarker concentration (*n* = 295). Models adjusted for maternal age, education, race and ethnicity, and baseline BMI.

**Figure 5:**
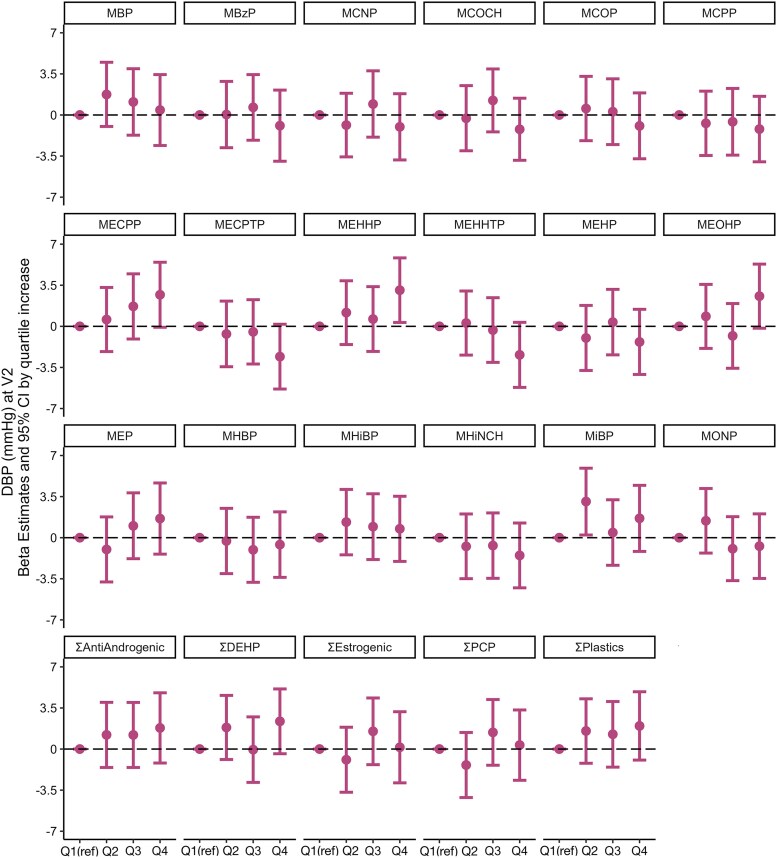
Single pollutant models of DBP at visit 2. Associations of log_2_-transformed and specific gravity corrected urinary biomarker concentrations with diastolic blood pressure at visit 2 expressed as the difference in mean DBP (mmHg) per quartile increase in biomarker concentration (*n* = 280). Models adjusted for maternal age, education, race and ethnicity, and baseline BMI.

**Figure 6:**
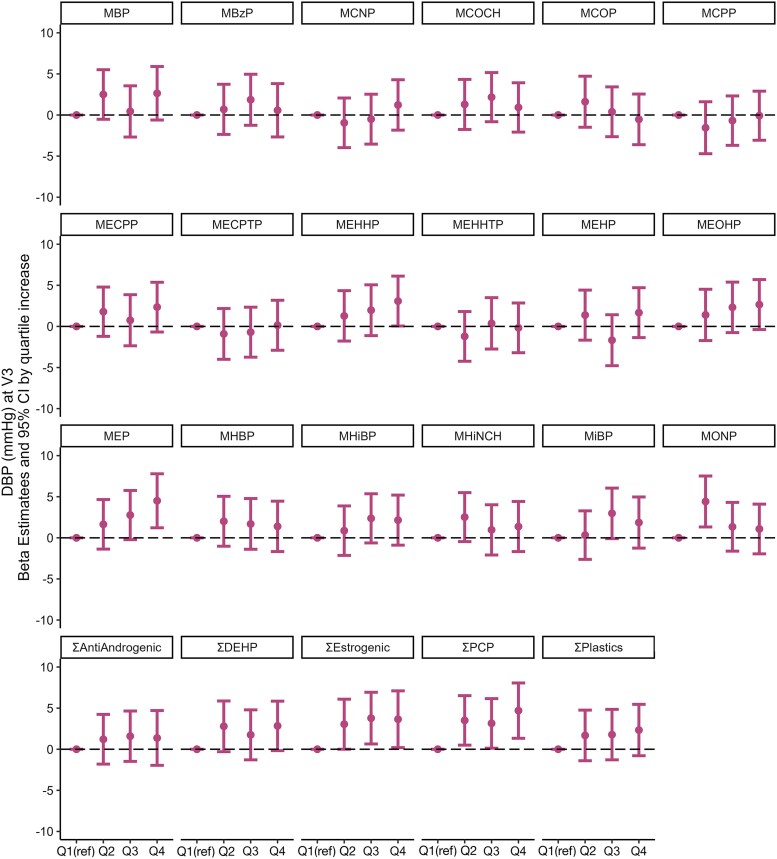
Single pollutant models of DBP at visit 3. Associations of log_2_-transformed and specific gravity corrected urinary biomarker concentrations with diastolic blood pressure at visit 3 expressed as the difference in mean DBP (mmHg) per quartile increase in biomarker concentration (*n* = 266). Models adjusted for maternal age, education, race and ethnicity, and baseline BMI.

DBP results in Figs. 4-6, Table S4 [19], and Fig. S5 [19] show several positive cross-sectional associations of urinary phthalate/DINCH biomarkers at pregnancy visits V1-V3 with DBP [19]. Specifically, we observed positive associations between biomarker concentrations at visit 1 (MBzP and MCPP), visit 2 (MEHHP and MiBP), and visit 3 (MEHHP, MEP, MONP, ΣEstrogenic, and ΣPCP).

At V1, among individual metabolites, the associations of MBzP (Q4 vs Q1: β = 3.94 mmHg, 95% CI: 1.02, 6.86, Per doubling: β = 0.66, 95% CI: 0.03, 1.29), as well as MCPP (Q2 vs Q1: β = 2.88 mmHg, 95% CI: 0.13, 5.62) was associated with higher mean DBP (Fig. 4).

At V2, higher concentrations of MEHHP at Q4 (Q4 vs Q1: 95% CI: 0.33, 5.83) and MiBP at Q2 (Q2 vs Q1: 95% CI: 0.23, 5.92) were both associated with a statistically significant increase of 3.1 mmHg for DBP, although no positive trend for per doubling or across quartiles was observed. Conversely, each doubling of MECPTP concentration was inversely associated with lower DBP at V2 (β = −0.59 mmHg, 95% CI: −1.08, −0.10) (Fig. 5).

At V3, we found the high molecular weight phthalate metabolites, MEHHP and MONP, were both linked with higher mean DBP. Specifically, concentrations of MEHHP (Q4 vs Q1: β = 3.08 mmHg, 95% CI: 0.05, 6.12, per doubling: β = 0.94 mmHg, 95% CI: 0.06, 1.83) and MONP (Q2 vs Q1: β = 4.42 mmHg, 95% CI: 1.32, 4.31) were associated with increases of DBP. We also found positive associations between higher MEP concentrations (Q4 vs Q1: β = 4.51, 95% CI: 1.22 mmHg, 7.79, per doubling: β = 1.03 mmHg, 95% CI: 0.30, 1.76) and higher mean DBP at V3. Moreover, we found positive associations across quartiles of ΣEstrogenic and ΣPCP concentrations with 3-5 mmHg higher mean DBP levels compared to Q1. Each doubling of ΣEstrogenic and ΣPCP concentrations also was associated with increases in DBP at V3 of 1.05 mmHg (95% CI: 0.14, 1.96) and 1.28 mmHg (95% CI: 0.37, 2.20), respectively (Fig. 6).

### Associations between urinary metabolite concentrations—individual and summary measures—with HDP risk

Results from log-binomial regression models between urinary biomarker concentrations at pregnancy visit 1 (V1) and HDP risk are shown in Table S5 and Fig. S6 [19]. At V3, participants with moderate MHiNCH concentrations (Q2) had more than 3-fold higher adjusted risk of HDP (adjusted RR = 3.30, 95% CI: 1.07, 10.21) compared to the lowest group (Q1). We also observed inverse associations of MBzP concentrations in Q3 (vs Q1) with risk of HDP (adjusted RR = 0.32, 95% CI: 0.10, 0.98). No other associations between HDP and urinary metabolites were observed in adjusted models.

### Associations between metabolite mixtures, blood pressure, and HDP risk

**Figure 7:**
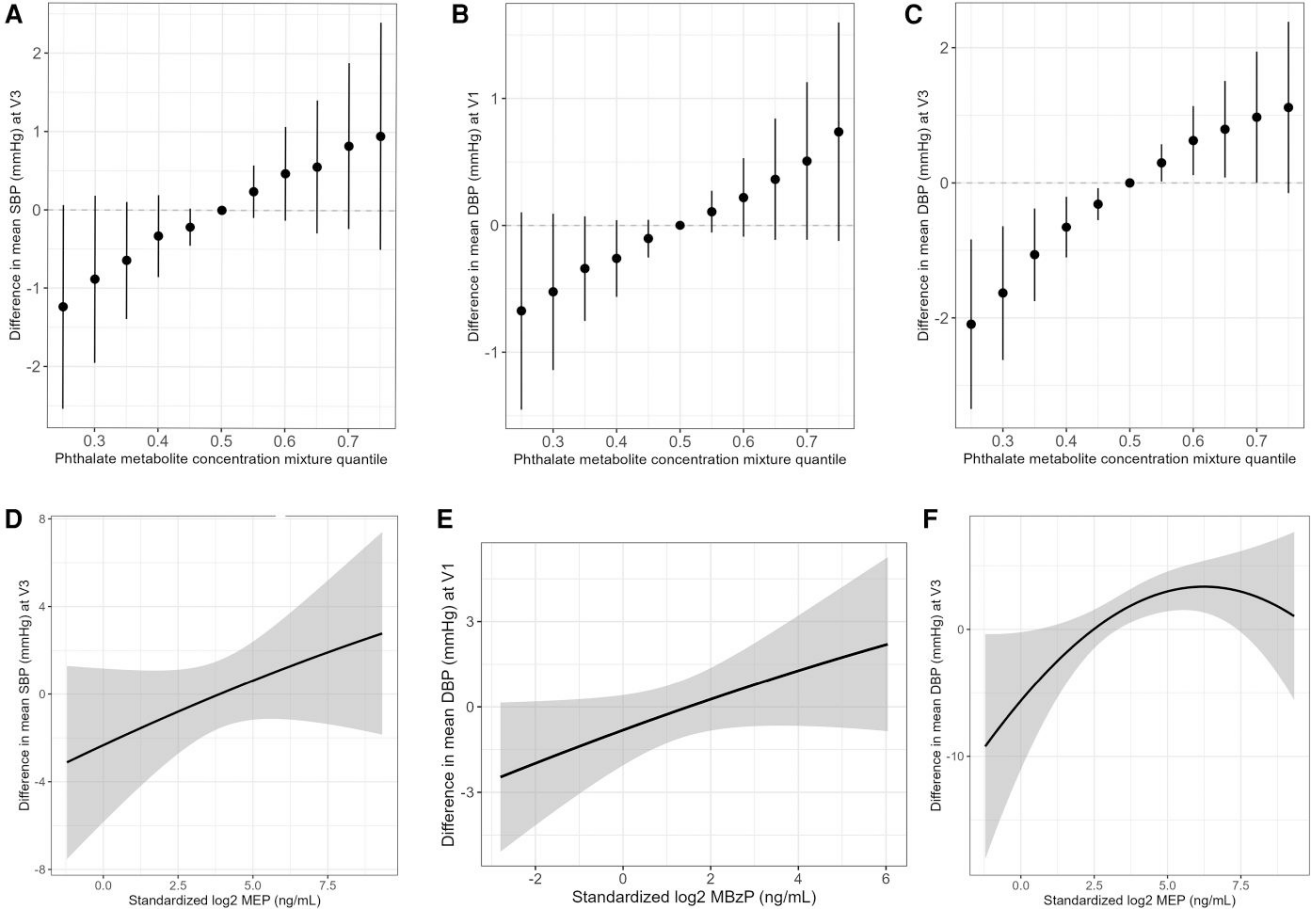
Bayesian kernel machine regression overall mixture results of SBP and DBP at visit 3. Overall associations of urinary phthalate/DINCH metabolite mixture and gestational blood pressure estimated from Bayesian Kernel Machine Regression: (A) SBP levels at V3 (B) DBP levels at V1, and (C) DBP levels at V3, comparing all metabolite concentrations are held at a specific percentile compared to when all metabolites are held at their median values. Univariate exposure-response functions: (D) MEP and SBP at V3 (E) MBzP and DBP at V1 (F) MEP and DBP at V3, while holding all other metabolite concentrations at their median concentrations. Models adjusted for age at consent, education, race and ethnicity, and baseline BMI. Abbreviations: BMI, body mass index; DBP, diastolic blood pressure; MEP, monoethyl phthalate; MBzP, monobenzyl phthalate; SBP, systolic blood pressure.

Our Bayesian kernel machine regression analysis for the overall effects of the metabolite mixture and the estimated differences in gestational blood pressure (mmHg) when all metabolites are held at the 25th to 75th percentiles, compared to when all metabolite concentrations are held at the median are shown in Fig. 7. We observed positive trends between the overall metabolite mixture on (1) SBP at V3, (2) DBP at V1, and (3) DBP at V3. However, we did not observe any meaningful independent or joint associations between the overall metabolite mixture and SBP levels at V2 and V3, and DBP at V2 (Fig. S7, Tables S10 and S11) [19].

From the univariate response curve, MEP concentrations showed a positive association with DBP, with a potential leveling off of the association at the upper exposure biomarker concentrations (Fig. 7F). Specifically, a change in MEP concentrations from the 25th to 75th percentile at V3 was associated with 2.44 mmHg increase in DBP levels (95% credible interval: 0.73, 4.15), while all other metabolites were held at their median levels. MEP concentrations were found to be the most important contributor at V3, with posterior inclusion probabilities of 0.35 (Table S12) [19]. We did not observe any significant associations for metabolite mixtures with risk of HDP (Fig. S8, Tables S13 and Table S14) [19].

### Sensitivity analysis

Overall, the positive associations observed in our main analysis were slightly higher in magnitude after excluding baseline BMI from single pollutant models of SBP and DBP (Tables S6 and S7) [19]. The only exceptions were associations for Q4 vs Q1 associations between MEP with SBP levels at V3 and MEHHP with DBP levels at V2, which became attenuated and no longer significant. We observed stronger positive associations for MEP and MCOP concentrations and mean SBP at V1 (Q3 vs Q1 for MEP: β = 4.36 mmHg, 95% CI: 0.60, 8.12 and Q4 vs Q1 for MCOP: β = 3.62 mmHg, 95%CI: 0.01, 7.23, respectively). At V3, positive associations between MBP, MCPP, and MEOHP metabolites and mean DBP also were strengthened, reaching statistical significance upon excluding BMI.

In our analysis restricted to nulliparous individuals, we observed positive relationships between several biomarker concentrations and SBP levels at V1, and inverse relationships for both SBP and DBP levels at V3 (Tables S8 and S9) [19]. Notably, we found a positive trend with low molecular weight metabolites (MBP, MHBP, MiBP, MHiBP), including ΣAnti-androgenic, ΣEstrogenic and ΣPCP and SBP at V1. Conversely, an inverse trend was observed between several high molecular weight metabolites (MCNP, MCPP, MECPP, and MONP) with SBP and DBP at V3. Specifically, per doubling increases of urinary concentrations of MBP were associated with higher SBP levels at V1 (β = 1.28 mmHg, 95% CI: 0.02, 2.53), whereas MCOP concentration was associated with lower SBP levels at V3 (β = −1.90 mmHg, 95% CI: −3.58, −0.22). From our main findings, each doubling of MEP concentrations was associated with both higher SBP and DBP at V3, however, among nulliparous participants these associations were weaker (SBP: β = 0.69 mmHg, 95% CI: −0.90, 2.29; DBP: β = 0.87, 95% CI: 0.14, 2.14). When we excluded participants taking anti-hypertensive medication (*n* = 14) and SPRING participants (*n* = 15) results were similar to our main findings (data not shown). Finally, we report several marginal, statistically significant associations before multiple testing correction, however, none remained significant after false discovery rate adjustment (*q* < 0.05).

## Discussion

This study examined associations of individual and mixtures of urinary phthalate and the replacement chemical DINCH biomarkers concentrations during pregnancy with SBP and DBP measures in a prospective, longitudinal pregnancy cohort based in Boston, MA. Our main findings suggest that higher urinary concentrations of MEP, ΣEstrogenic, and ΣPCP were cross-sectionally associated with modest to moderate elevations in SBP and DBP in early and late pregnancy. Furthermore, excluding baseline BMI strengthened positive associations between several urinary metabolite phthalate concentrations and blood pressure across pregnancy (eg, MEP). Among nulliparous participants, we observed a positive trend of low molecular phthalate metabolite concentrations linked with higher SBP at V1 (eg, MBP, MiBP). From Bayesian kernel machine regression, the overall metabolite mixture was positively associated with DBP in late pregnancy, with MEP identified as the most important relative contributor to the association. One metabolite of the phthalate replacement DINCH was associated with an increased risk of HDP, while MBzP was associated with a decreased risk of HDP. However, these findings were tempered by the fact that associations were often only observed for specific quartiles, and did not consistently demonstrate a strong dose response pattern.

### Previous studies—gestational SBP and DBP results

In the present study, MEP, ΣEstrogenic, and ΣPCP showed the most consistent positive associations with blood pressure, particularly in early and late pregnancy. Previous studies evaluating phthalates and blood pressure measurements in pregnancy are consistent with our findings, with MEP being positively associated with blood pressure. Notably, 2 cohorts based in the United States reported positive associations in early pregnancy with gestational blood pressure measurements [32, 35, 39]. Studies that reported null or inverse associations took place outside of the United States and urine samples were collected during an earlier time period (samples were collected between 2007 and 2015) [14]. This, difference in background exposures may explain differences in associations due to varying regulatory standards and laws governing the inclusion of DEP, the precursor of MEP, particularly in personal care products [40-42]. The present study is one of the first to evaluate ΣEstrogenic and ΣPCP summary measures and gestational blood pressure. These findings suggest that PCPs are an important source of exposure and that they could operate through an estrogenic pathway.

Other metabolites were associated with gestational blood pressure. For example, in early pregnancy, we observed positive associations of MCNP with SBP and MBzP, and MCPP with DBP in early pregnancy. Visit 2, which occurred at median of 19 weeks of gestation, preceded the well-documented mid-pregnancy decline in blood pressure between 20 and 26 weeks, likely driven by systemic vasodilation and hormonal changes, which may reflect the attenuated associations observed at this visit [43, 44]. In mid-pregnancy, we saw mixed results for MECPTP, MEHHTP, MEHHP, and MiBP with DBP. For late pregnancy, we saw associations of MHiNCH with SBP, along with MCPP and MONP, and DEHP metabolites with DBP. The association between MBzP and blood pressure was consistent with the PROGRESS study, which took place in Mexico City, where each doubling of MBzP concentration in the second trimester was associated with a 0.5 mmHg higher DBP in the third trimester, despite lower median exposure biomarker concentrations than the present study [40]. Positive associations were seen for MBzP and DBP among participants in the HOME Study (Ohio, United States) when assessing blood pressure prior to 20 weeks of gestation. However, these associations were not seen when evaluating MBzP and DBP assessed after 20 weeks [39, 45]. Also, previous studies did not find associations for the other metabolites and gestational blood pressure. These differences in associations could be attributed to differences in urinary biomarker concentrations, as well as the study design. In particular, biomarker concentrations of phthalate replacement chemicals such as DINCH have increased over the past decade. In addition, we evaluated the cross-sectional associations between these non-persistent chemicals and blood pressure measured at the same time point of collection—a time period in gestation that may be particularly relevant as it relates to the biological impact of these chemicals. Future studies may be needed to consider key timing of the exposure assessment as it relates to phthalates and replacement chemicals and their impact on gestational blood pressure.

In a previous study of metabolite mixtures and pregnancy blood pressure, authors reported a positive association between overall phthalate mixtures and DBP in the second trimester and third trimester [40]. Our Bayesian kernel machine regression results also showed positive associations between metabolite mixtures and higher mean DBP, particularly for late pregnancy. Moreover, the positive results from single pollutant models observed between MEP concentrations and DBP at V3 were consistent with findings from the PROGRESS study [40]. Among the 18 urinary metabolites evaluated, MEP emerged as the strongest contributor to these visit-specific associations. Our findings from both phthalate/DINCH mixtures and single-exposure analyses suggest that monitoring and identifying sources (eg, personal care products) may be key to reducing time-sensitive phthalate or DINCH exposure during pregnancy.

Finally, recent data from pooled analyses found positive associations between HDP and specific urinary phthalate metabolite concentrations. However, we could not corroborate these findings. Two separate meta-analyses showed urinary MEP was associated with 12% and 37% higher odds of HDP, respectively [13, 14]. Another pooled analysis of ECHO cohorts (*n* = 3430) based in the United States, showed that other phthalate metabolites, MCPP and MBzP, were positively associated with nominal increases in the odds of HDP [15]. In our study, moderate maternal urinary concentrations of the DINCH metabolite MHiNCH in Q2 were associated with higher risk of HDP, compared to those in Q1. While higher MBzP concentrations were associated with a decreased risk of HDP. No other associations were seen for metabolites and HDP in the present study.

Overall, we observed stronger associations with continuous blood pressure measures than with the dichotomous HDP outcome. The inverse association between MBzP and HDP is likely a chance finding due to multiple comparisons and chance reflected by the wide confidence intervals. Differences in findings between blood pressure and HDP may reflect reduced statistical power for the binary outcome of HDP, as well as random variation due to small sample size, including between our full cohort and nulliparous subsample. In particular, parous mothers tend to be older and have different risk factors including reproductive histories that may affect their susceptibility to EDCs. HDP was a secondary analysis, as the present study had limited power to assess this binary outcome given the small number of individuals with this condition, so these results should be interpreted with caution.

### Mechanisms of blood pressure regulation

In a healthy pregnancy, blood pressure declines from 20 to 26 weeks of gestation, then rises to pre-pregnancy levels in late pregnancy. Phthalates and their replacements may disrupt this pattern in several ways. Phthalates and potentially their replacement metabolites are hypothesized to impair endothelial health, which may alter maternal blood pressure through changes in arterial resistance or elasticity [46]. These chemicals have been shown to stimulate pro-inflammatory cytokines production, which may contribute to increase vascular resistance, atherosclerosis and plaque buildup, vascular remodeling, and alter hormones involved in the regulation of blood pressure [47-50]. Phthalates and their replacement chemicals may influence the regulation of blood pressure by impeding renal function, activating the sympathetic nervous system, and stimulating the hormone thyroxin [51-54]. Further, outside of pregnancy and in non-pregnant women, estrogen exposure from certain combined oral contraceptives has been associated with higher blood pressure [55]. Although the biological mechanisms through which phthalates or their replacements influence blood pressure regulation remain unclear, growing epidemiological evidence suggests that environmental exposure to phthalates may be an important risk factor for elevated blood pressure and HDP. Recent data also indicate that modest elevations of blood pressure, even those below diagnostic thresholds for HDP, may signal cardiovascular maladaptation of vascular and endocrine pathways that regulate blood pressure, increasing the risk of maternal cardiovascular disease later in life [2, 56].

### Strengths

In this prospective cohort, pregnant participants were recruited early in gestation to examine concentrations of urinary metabolites of phthalates and their replacement chemicals to capture recent maternal exposures (2016-2020). Few studies have examined time-varying endocrine disrupting chemical biomarkers and blood pressure measurements. By collecting 3 urine samples at 3 distinct visits during pregnancy, we were able to assess visit-specific phthalate and other EDC biomarker associations and their concurrent associations with blood pressure at each time point, evaluating potential sensitive windows of exposure. Across previous studies, there were methodological differences in the reporting of gestational age and HDP diagnosis. We conducted a prospective analysis to ensure biomarker concentrations were measured before HDP diagnosis (<20 weeks). We also evaluated 5 summary biomarkers reflecting correlation, source of exposure, and shared mechanistic activity.

### Limitations

Phthalates and their replacement chemicals are rapidly metabolized and largely excreted in the urine. However, research shows that phthalates can accumulate in the kidneys to exert nephrotoxic effects [57]. As such, individuals with kidney disease may metabolize these chemicals differently than those without this condition. Importantly, both renal and angiotensin systems are involved in the regulation of blood pressure and development of HDP [58]. In our study, no participants reported having renal disease or family history of renal disease, and we lacked direct measures of rental function (eg, eGFR), however, because kidney disease is relatively uncommon among 18- to 44-year-olds in the United States (approximately 6%), we do not anticipate any substantial undiagnosed kidney disease in ERGO [19]. The other limitations of this study include a small sample size in a population that has a higher level of educational attainment compared to the average population of the United States; however, the educational attainment is similar to that of the Boston area. Compared to the general pregnant population of the United States, the overall HDP prevalence was higher in our study, suggesting risk factors for HDP such as older maternal age may have differed. We used medical record abstraction to obtain blood pressure measurements from multiple sites. The majority of participants had urine collected and blood pressure measured on the same day. In a subset of participants (n = 116, 34%) blood pressure was measured shortly after urine collection, approximately 3-4 days later (mean: 0.5 weeks; median: 0.4 weeks). Given that there were some differences in blood pressure methods among sites, variability is plausible; however, we expect this to contribute to non-differential misclassification as it is unlikely that differences in blood pressure methods by hospital were associated with metabolite concentrations. We imputed concentrations below the limit of detection using limit of detection/√2, which may bias estimates when modeling continuous exposures; however, we also analyzed exposure biomarker concentrations by quartiles to address this limitation. Single spot urine samples were collected at each visit; thus, our visit-specific measures may not fully capture day-to-day variability of exposure to phthalates and their replacements within each window, thus, findings should be interpreted with caution. Finally, it is probable that pregnant women at risk for HDP, undergo more frequent routine blood pressure measurements during prenatal visits. However, we used blood pressure measurements that were taken closest to the time following when urine was collected to assess the relevant time window and account for the relatively short half-life of the target chemicals.

## Conclusion

Our study found that during certain windows of pregnancy, individual associations of MEP, ΣEstrogenic, and ΣPCP were cross-sectionally associated with higher SBP and DBP. Associations were modest, but strongest in later pregnancy. These findings suggest that diethyl phthalate, the parent chemical of MEP, which is highly prevalent and commonly used in personal care products, could contribute to elevations in blood pressure in pregnancy. However, additional studies are needed to further elucidate this finding, particularly as it relates to the potential for an underlying estrogenic mechanism. Other phthalates and their replacements may also be important and warrant further evaluation. If replicated, the current study suggests that reduction in phthalate exposures, particularly from those found in personal care products, could reduce risk of cardiovascular disease related outcomes in populations of pregnant women.

## Data Availability

Some or all datasets generated during and/or analyzed during the current study are not publicly available but are available from the corresponding author on reasonable request.

## Abbreviations

ΣAnti-Androgenic: molar sum of metabolites of parent chemicals with anti-androgenic properties
BzBP: benzyl butyl phthalate
BMI: body mass index
DBP: diastolic blood pressure
DiBP: di-isobutyl phthalate
DnBP: di-n-butyl phthalate
DnOP: di-n-octyl phthalate
DEHP: di-2-ethylhexyl phthalate
ΣDEHP: molar sum of di(2-ethylhexyl) phthalate metabolites
DEHTP: di-2-ethylhexyl terephthalate
DEP: diethyl phthalate
DiDP: di-isodecyl phthalate
DiNP: di-isononyl phthalate
DINCH: di(isononyl) cyclohexane-1,2-dicarboxylate
EDCs: endocrine disrupting chemicals
ERGO: Environmental Reproductive and Glucose Outcomes study
ΣEstrogenic: molar sum of metabolites of parent chemicals with estrogenic properties
HDP: hypertensive disorders of pregnancy
mmHg: millimeters of mercury
MBP: mono-n-butyl phthalate
MBzP: monobenzyl phthalate
MCOCH: cyclohexane-1,2-dicarboxylic acid-mono(carboxyoctyl) ester
MCNP: monocarboxyisononyl phthalate
MCOP: monocarboxyoctyl phthalate
MCPP: mono(3-carboxypropyl) phthalate
MECPP: mono(2-ethyl-5-carboxypentyl) phthalate
MECPTP: mono(2-ethyl-5-carboxypentyl) terephthalate
MEHHP: mono(2-ethyl-5-hydroxyhexyl) phthalate
MEHHTP: mono(2-ethyl-5-hydroxyhexyl) terephthalate
MEHP: mono(2-ethylhexyl) phthalate
MEOHP: mono(2-ethyl-5-oxohexyl) phthalate
MEP: monoethyl phthalate
MHBP: mono-hydroxybutyl phthalate
MHiBP: mono-hydroxy-isobutyl phthalate
MHiNCH: cyclohexane-1,2-dicarboxylic acid-monohydroxy-isononyl ester
MiBP: mono-isobutyl phthalate
MONP: monooxononyl phthalate
PCPs: personal care products
ΣPCP: molar sum of metabolites of parent chemicals used in personal care products
ΣPlastics: molar sum of metabolites of parent chemicals used in plastics
RR: risk ratios
SBP: systolic blood pressure
